# Reproduction of ectoparasitic mites in a coevolved system: *Varroa* spp.—Eastern honey bees, *Apis cerana*

**DOI:** 10.1002/ece3.7038

**Published:** 2020-12-01

**Authors:** Shuai Wang, Zheguang Lin, Gongwen Chen, Paul Page, Fuliang Hu, Qingsheng Niu, Xiaoling Su, Panuwan Chantawannakul, Peter Neumann, Huoqing Zheng, Vincent Dietemann

**Affiliations:** ^1^ College of Animal Sciences Zhejiang University Hangzhou China; ^2^ College of Animal Science and Technology Yangzhou University Yangzhou China; ^3^ Swiss Bee Research Center Agroscope Bern Switzerland; ^4^ Apiculture Science Institute of Jilin Province Jilin China; ^5^ Jinhua Academy of Agricultural Sciences Jinhua China; ^6^ Department of Biology and Environmental Science Research Center (ESRC) Faculty of Science Chiang Mai University Chiang Mai Thailand; ^7^ Vetsuisse Faculty Institute of Bee Health University of Bern Bern Switzerland; ^8^ Department of Ecology and Evolution University of Lausanne Lausanne Switzerland

**Keywords:** honey bee, host shift, infestation, reproduction, *Varroa* mites

## Abstract

Parasite host shifts can impose a high selective pressure on novel hosts. Even though the coevolved systems can reveal fundamental aspects of host–parasite interactions, research often focuses on the new host–parasite relationships. This holds true for two ectoparasitic mite species, *Varroa destructor* and *Varroa jacobsonii*, which have shifted hosts from Eastern honey bees, *Apis cerana*, to Western honey bees, *Apis mellifera*, generating colony losses of these pollinators globally. Here, we study infestation rates and reproduction of *V. destructor* and *V. jacobsonii* haplotypes in 185 *A. cerana* colonies of six populations in China and Thailand to investigate how coevolution shaped these features. Reproductive success was mostly similar and low, indicating constraints imposed by hosts and/or mite physiology. Infestation rates varied between mite haplotypes, suggesting distinct local co‐evolutionary scenarios. The differences in infestation rates and reproductive output between haplotypes did not correlate with the virulence of the respective host‐shifted lineages suggesting distinct selection scenarios in novel and original host. The occasional worker brood infestation was significantly lower than that of drone brood, except for the *V. destructor* haplotype (Korea) from which the invasive lineage derived. Whether mites infesting and reproducing in atypical intraspecific hosts (i.e., workers and queens) actually predisposes for and may govern the impact of host shifts on novel hosts should be determined by identifying the underlying mechanisms. In general, the apparent gaps in our knowledge of this coevolved system need to be further addressed to foster the adequate protection of wild and managed honey bees from these mites globally.

## Graphical abstract

Here, we study brood infestation and reproduction of *Varroa destructor* and *Varroa jacobsonii* haplotypes in 185 colonies of six populations of the original host *Apis cerana* in China and Thailand to describe the product of coevolution and to identify traits facilitating host shifts. Infestation rates varied between mite haplotypes, suggesting distinct local co‐evolutionary scenarios, but reproductive success was similar, indicating biological constraints imposed by the host. Drone brood infestations were orders of magnitude higher compared to worker brood.

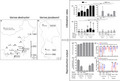

## 1 INTRODUCTION

Parasites are among the strongest selection forces shaping the evolution of their hosts (Anderson, 1994). Their impact is further exacerbated when they can shift to host species never encountered before, which can negatively affect host populations (Kumschick et al., 2015; Pimentel et al., 2005). A better knowledge of the mechanisms allowing for host shifts and driving coevolution between parasites and original as well as new hosts could help mitigate the parasites’ detrimental effects (Kolar & Lodge, 2001; Woolhouse et al., 2005). Honey bees are a good model species to investigate such mechanisms because trade activities exploiting the pollination service and hive products they provide (Gallai et al., 2009; Huang et al., 2018; Kleijn et al., 2015; Klein et al., 2007; Potts et al., 2010) have led to colonies of difference species being brought into contact. As a result, natural barriers separating species have been overcome, allowing parasites to shift host. For instance, two species of the ectoparasitic mite genus *Varroa* parasitizing *A. cerana* (*Varroa destructor* and *Varroa jacobsonii*) shifted to *A. mellifera* (Anderson, 2008; Crane, 1968; Roberts et al., 2015; Rosenkranz et al., 2010; Sakai & Okada, 1973), which was not parasitized by this mite genus in its original distribution range.

*Varroa* spp. mites parasitize the host's immature brood by entering the cells in which the latter develop. This occurs hours before adult workers seal the cells using wax, ahead of host pupation. Shortly after their confinement under the wax cap, mites start reproducing, which is interrupted by host emergence out of the cell (Nazzi & Le Conte, 2016). In the original host, *Varroa* spp. only reproduce on drone brood present during a short period of the colony's yearly life cycle. In contrast, and probably due to the lack of coevolution, in the new host, reproduction of the invasive *V. destructor* occurs both in the drone brood and in the worker brood, which is produced over several months (Anderson & Sukarsih, 1996; Boot et al., 1997; Koeniger et al., 1981; Tewarson et al., 1992). Reproduction on worker brood allows for large increases of the parasite population in the colonies and results in large‐scale colony losses of *A. mellifera* (Rosenkranz et al., 2010).

Given its worldwide spread, the host‐shifted *V. destructor* lineage has become one of the most lethal pests for *A. mellifera* (Dietemann et al., 2012; Li et al., 2012; Neumann & Carreck, 2010; Potts et al., 2010; Roberts et al., 2015; Smith et al., 2013; Steinhauer et al., 2018). Accordingly, much research has focused on the interactions between *A. mellifera* and *V. destructor*, whereas little interest has been dedicated to its relationship with *A. cerana* (Dietemann et al., 2012; Rosenkranz et al., 2010). The same holds true for *V. jacobsonii*, whose global negative impact is lower (Oldroyd, 1999). Studies of *V. jacobsonii* and *V. destructor* in *A. cerana* report natural infestation rates of brood or adults and occasionally mite fertility, but there is little information on mite fecundity and reproductive success (Anderson & Sukarsih, 1996; Boot et al., 1997; Koeniger et al., 1981, 1983; Rath & Drescher, 1990; Rosenkranz et al., 1993; Tewarson et al., 1992). These parameters affect infestation rates and hence potential damage inflicted to host colonies. Their quantification is thus fundamental to our understanding of the mechanisms sustaining the coevolved and balanced host–parasite relationship (Locke, 2016) and to our ability to limit the damage generated by the parasite by, for example, selecting for host resistance traits (Dietemann et al., 2012). To fill the lack of data on infestation and reproduction of *Varroa* spp. on their original host, we measured adult and brood infestation rates, as well as several parameters of reproductive output of *V. destructor* and *V. jacobsonii* mites naturally infesting *A. cerana*. To consider possible variations in co‐evolutionary scenarios leading to different reproductive strategies between populations (Schluter, 2009; Thompson, 2005) and obtain a representative picture of the host–parasite interactions between *Varroa* spp. and its original host, we screened *A. cerana* colonies in several regions in China and Thailand. In Thailand, *A. cerana indica* populations are infested by three *V. jacobsonii* haplotypes: North Thai infesting the Mainland host haplotype in the north of the country below 1,000 m altitude and Samui on Samui Island and Malay in the south, infesting the Sundaland host haplotype (Dietemann et al., 2019; Hepburn et al., 2001; Radloff et al., 2010; Rueppell et al., 2011; Warrit et al., 2006). In Eastern China, we screened *A. cerana cerana* populations infested by three *V. destructor* haplotypes: Japan in the north, Korea in the central region, and China in the south (Anderson & Trueman, 2000; Hepburn et al., 2001; Navajas et al., 2010; Radloff et al., 2010; Zhou et al., 2004; Z Lin, S Wang, P Neumann, G Chen, P Page, L Li, F Hu, H Zheng & V Dietemann, unpublished data). To broaden the range of *Varroa* spp.*–Apis* spp. systems considered, we compared our results with those recently reported for the closely related species *Varroa underwoodi* (Wang et al., 2019) and with previous literature on the *V. destructor* lineage reproducing *on A. mellifera*. We discuss how the differences in infestation rates and reproductive output of the haplotypes studied contribute to our understanding of host–parasite relationship in the *Varroa* spp.*–Apis* spp. system.

## 2 MATERIALS AND METHODS

### 2.1 Sampling

**Figure 1:**
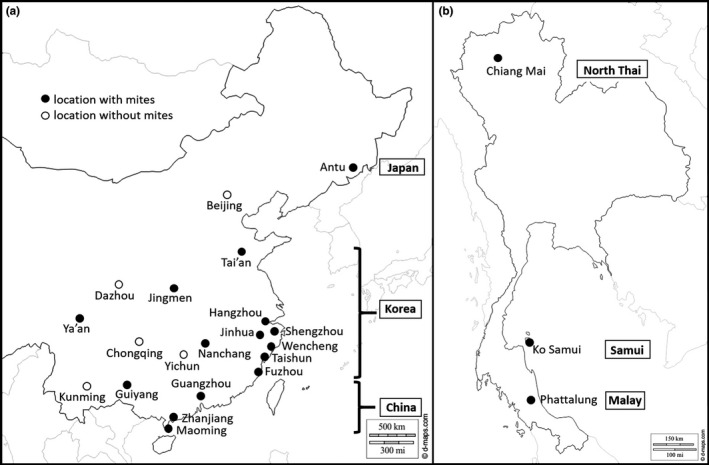
Sampling locations and haplotypes of (a) *Varroa destructor* in Eastern China and (b) *Varroa jacobsonii* in Thailand

From 2013 to 2018, during local spring to early summer when colonies produce drones ahead of the swarming and mating period, capped brood cells were screened for mite infestations in managed colonies of *A. cerana*. These colonies were not treated with any chemical acaricide. The sampling regions covered the natural distribution ranges of the *V. destructor* China, Japan, and Korea haplotypes in China (Navajas et al., 2010; Z Lin, S Wang, P Neumann, G Chen, P Page, L Li, F Hu, H Zheng & V Dietemann, unpublished data) and of *V. jacobsonii* North Thai, Malay, and Samui haplotypes in Thailand (Dietemann et al., 2019; Warrit et al., 2006). In Eastern China, *V. destructor* mites were collected from 151 colonies in 20 regions (Table S1). As many drone cells as possible were screened for mites in each of these colonies, and when possible, worker cells were also screened (in 28 of these colonies). In Thailand, *V. jacobsonii* mites were collected from 34 colonies in three regions. Drone cells were screened in 31 colonies and worker cells in 18 colonies. Three of these colonies did not belong to the sample of 31 colonies used for drone cells screening because of drone brood unavailability. In total, 57,773 drone and 14,726 worker brood cells were screened for infestations with the two mite species (Figure 1; Table S1).

Razor blades were used to open the wax caps of each brood cell, and the larvae and pupae were extracted with forceps for cell inspection (Dietemann et al., 2013). In the case of infestation, mites were extracted using a fine paintbrush and the developmental stage and sex of the offspring were identified (Dietemann et al., 2013). Host developmental stages were also noted as follows: larvae, white eye pupae, pink eye pupae, purple eye pupae, yellow thorax pupae, gray thorax pupae, and pre‐emergence adults (Human et al., 2013).

For our measures, the mites were grouped according to their region of sampling that reflect their haplotypes (China: Japan, Korea, China; Zhou et al., 2004; Navajas et al., 2010; Z Lin, S Wang, P Neumann, G Chen, P Page, L Li, F Hu, H Zheng & V Dietemann, unpublished data; Thailand: North Thai, Samui, Malay, Warrit et al., 2006; Dietemann et al., 2019).

### 2.2 Parameters measured

Brood infestation rates were quantified as the percentage of infested brood cells per colony and of colonies per haplotype. The fertility rate of mites of each haplotype was calculated as the percentage of *Varroa* spp. foundress mites that produced at least one offspring (egg, protonymph, deutonymph, adult daughter or male) out of all foundresses found on host brood at prepupae and beyond, so that all individuals were at a developmental stage advanced enough to sustain mite reproduction (Dietemann et al., 2013). Mite fecundity (number of offspring produced) was quantified in cells containing hosts at and beyond the yellow thorax stage since all mite eggs have been laid at this stage. Foundresses infesting pre‐emergence hosts, which have produced a son and at least one mature daughter, were considered to be reproductively successful (Dietemann et al., 2013). The percentage of fertile and reproductively successful mites out of all foundresses was calculated. Total number of offspring produced and number of viable female offspring (presence of a male ensured mating) are reported for all foundresses (fertile and infertile, representing the population‐wide reproductive output) and for reproductively successful foundresses only (quantifying the individual reproductive output).

Only cases of single infestation were considered when calculating the fecundity and reproductive success, because per capita fecundity cannot be measured reliably when brood cells are infested by several foundresses (Dietemann et al., 2013). Single and multiple infestations were distinguished after identifying family composition within a cell and comparing it with a model of mite reproduction. This model was generated based on the number and developmental stages of the mites found in each infested cell (Table S2). The maximum number of offspring was determined based on the typical family composition observed in the cells screened. Thus, any cell containing more than this number was considered as multiply infested. The number of males was not considered in the identification of multiple infested cells since males could be missing (Boot et al., 1997; Martin, 1995; Martin & Kryger, 2002). This rule could not be used before the maximum of number of eggs had been laid by a foundress. For these cases, we compared the number of offspring at particular developmental stages with the model and considered stages with supernumerary offspring as indicative of multiple infestation. In cells in which female offspring did not yet reach the adult stage (before the purple‐eyed host stage), all dark female mites were considered as foundresses. Cases of infestation by multiple foundresses were counted to assess the frequency of such occurrence. The percentage of cells infested by single foundresses showing family patterns diverging from the models was calculated to determine the proportion of mites showing atypical reproduction.

Infestation rates of adult honey bees (*N* = 408–664 workers per colony) in 11 colonies in Hangzhou, China, were measured with icing sugar shakes. In Thailand, icing sugar clumped easily and was deemed unreliable to quantify adult infestation rate. Thus, we used a soapy water wash on 336–742 workers per colony in three colonies in Chiang Mai to quantify this rate. Both methods were implemented according to Dietemann et al. (2013). Data from the other regions were not collected given restricted access to worker samples.

### 2.3 Haplotyping

Given the high number of mites sampled, we chose to subsample for haplotype verification. Out of the 189 foundress mites of which reproductive output was assessed in Thailand, 35% were conserved to confirm the expected haplotype (Warrit et al., 2006). In China, a larger proportion (72%) of the 421 mites collected were haplotyped, because less detailed data were available in the literature to identify haplotypes in the region sampled (Anderson & Trueman, 2000; Navajas et al., 2010; Zhou et al., 2004). Haplotyping was performed according to Dietemann et al. (2019).

### 2.4 Statistical analyses

We tested whether the mite haplotypes, irrespective of species, differed in their brood infestation rates of colonies and cells as well as adult infestation rates. We also tested if they differed in percentage of fertile foundresses, of foundresses showing atypical reproduction patterns, and of foundresses showing reproductive success. The percentage of cells infested by multiple foundresses was also compared between haplotypes. Drone and worker brood were considered separately. For these comparisons, when expected cases had less than five counts, we used the Fisher test. When all expected cases had more than five counts, we used the Pearson chi‐square test (Siegel & Castellan, 1988). We also compared the haplotypes in terms of fecundity (total number of offspring produced) and reproductive success (number of viable daughters produced) on drone brood. For the analyses of the number of viable daughters and of the percentage of foundresses with reproductive success, only haplotypes with more than three data points were considered (i.e., individuals at the pre‐emergence stage). To compare these counts, we first determined whether the data were normally distributed with the Shapiro–Wilk method. In cases where the data were not normally distributed, we used nonparametric Kruskal–Wallis tests. When the overall comparison across haplotypes showed significant differences, we performed pairwise comparisons between haplotypes with the Pearson chi‐square or Fisher exact tests as described above for percentage parameters and with the Mann–Whitney U test for count parameters. Levels of significance were adjusted for multiple comparisons following Benjamini and Hochberg (1995), with a false discovery rate (FDR) of 0.07. This rate corresponds with one of the 15 comparisons made being a false positive. We chose this less conservative correction method due to the high number of comparisons and to reduce the chance for false negatives.

The correlations of the number of viable daughters (reproductive success) to fecundity (with successfully reproductive foundresses) and to brood infestation rates were analyzed by linear regressions methods among different haplotypes of the two mite species.

When comparing infestation rates and reproductive parameters between haplotypes, mites collected in the north of Thailand were considered as representative of the North Thai haplotype of *V. jacobsonii* despite the possible occasional occurrence of *V. destructor* in *A. cerana* colonies of this region (Dietemann et al., 2019).

The program SPSS Statistics 21.0 was used to perform these tests.

## 3 RESULTS

### 3.1 Infestation rates

**Figure 2:**
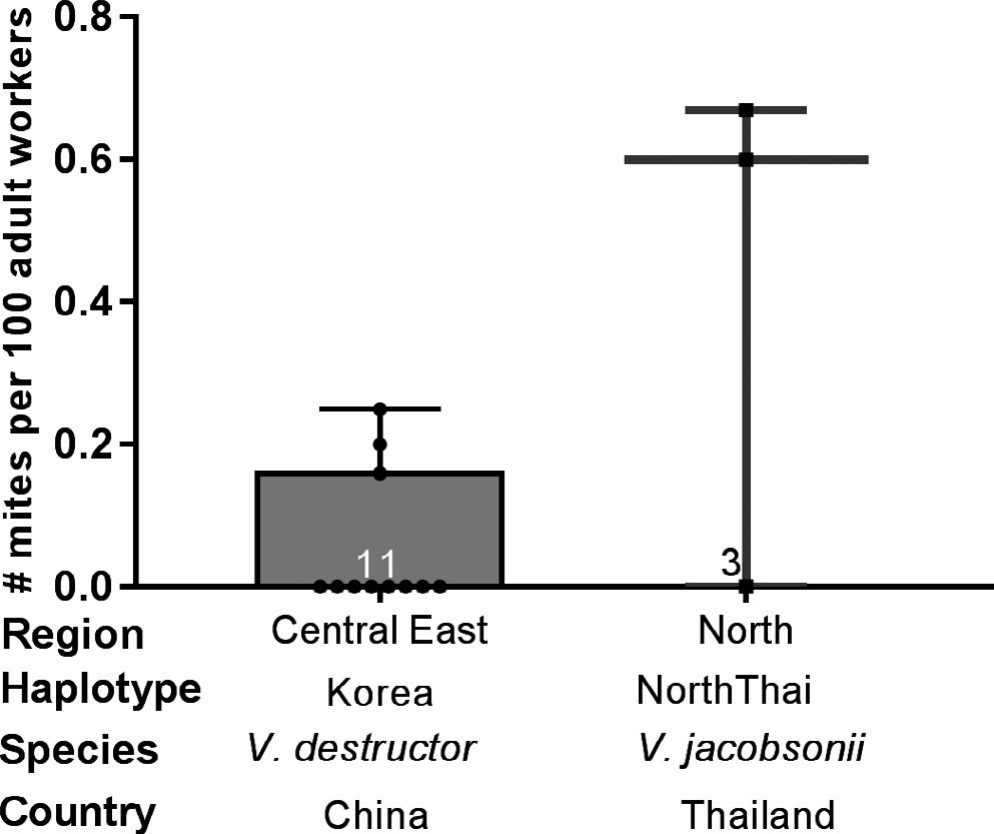
Infestation rates of *Apis cerana* adult worker by *Varroa destructor* Korea and *Varroa jacobsonii* North Thai mite haplotypes (black dots = data points, median = horizontal line, box = 25/75% quartiles, whiskers = min–max range). Sample size is given inor above the boxes

Adult worker infestation rates ranged between 0.00 and 0.67 mites per 100 workers in *V. destructor* Korea and *V. jacobsonii* North Thai, respectively, with no significant differences between any of the haplotypes (Mann–Whitney *U* test, *U* = 7.0, *p* = .09, Figure 2).

**Figure 3:**
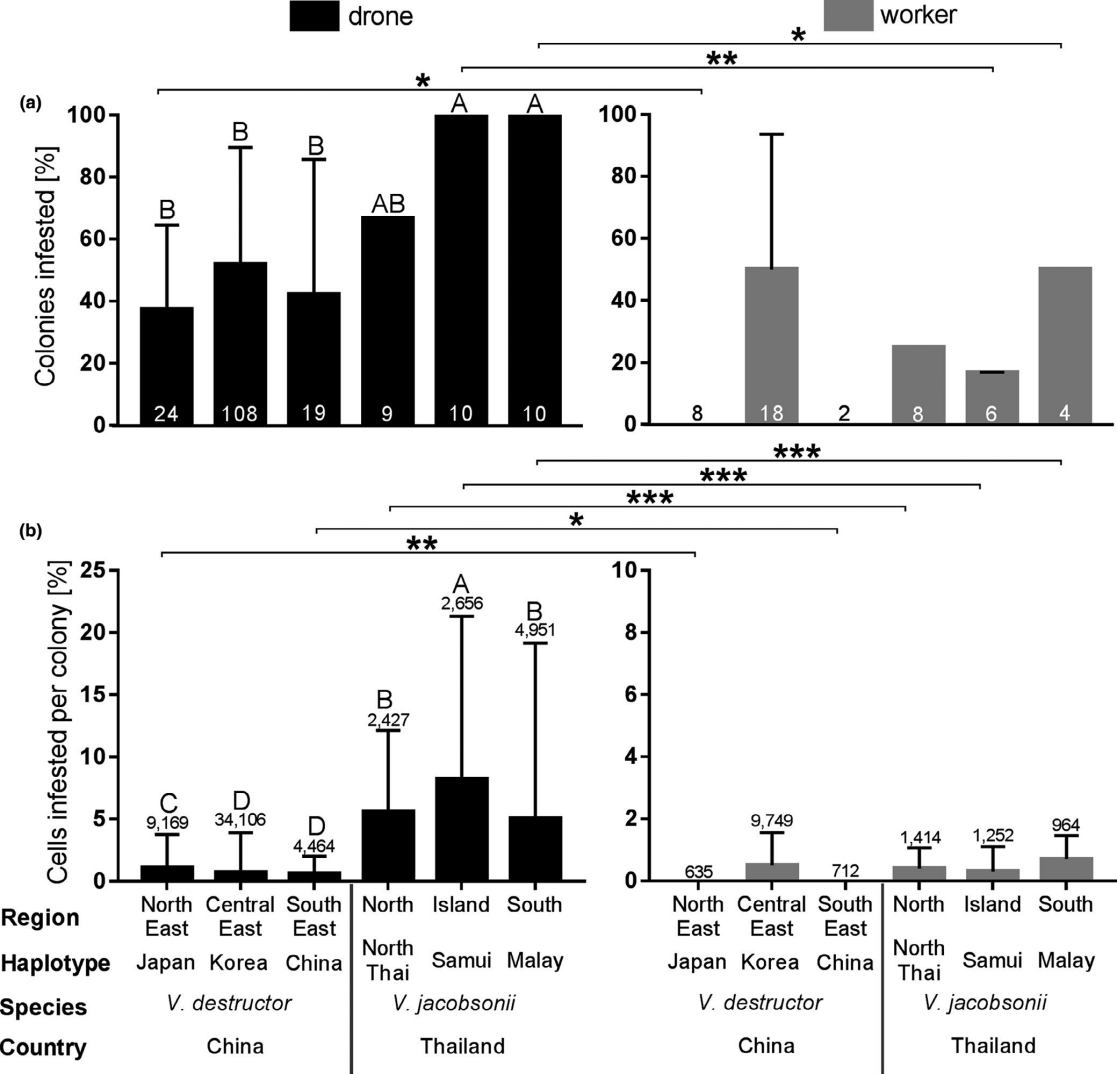
Infestation rates of *Apis cerana* drone and worker brood with *Varroa destructor* and *Varroa jacobsonii*. The percentage of infestations of each mite haplotype in China and Thailand are shown at (a) colony and (b) cell levels. The sample sizes are indicated within or above the bars. Asterisks indicate significant differences among all haplotypes using the Fisher exact test or Pearson chi‐square tests with FDR correction. *p < .05, **p < .01, ***p < .001. FDR, false discovery rate

In China, drone brood at some locations (in which one to five colonies were screened) were not infested. In contrast, in Thailand, drone brood of all colonies were infested. Overall, drone brood infestation by both *V. destructor* and *V. jacobsonii* haplotypes was common, ranging from 38% to 100% of the colonies screened. The percentage of colonies with infested drone brood differed significantly between some of the haplotypes: *V. jacobsonii* Samui and Malay were significantly more prevalent than all *V. destructor* haplotypes but not more prevalent than the North Thai haplotype of *V. jacobsonii*, which showed an intermediate value (Figure 3a; Table S3). Within colonies, drone brood average infestation rates ranged from 0.6% to 8.2%, with significantly lower rates for *V. destructor* compared to *V. jacobsonii* haplotypes (Figure 3b; Table S3). In China, the Japan haplotype of *V. destructor* infested significantly more drone brood than the Korea and the China haplotypes of this mite species. The latter two haplotypes did not differ significantly in the percentage of drone brood infested. In Thailand, the Samui haplotype of *V. jacobsonii* infested significantly more drone brood than the Malay and North Thai haplotypes. The infestation rates of the latter two haplotypes did not significantly differ from each other (Figure 3b; Table S3).

A maximum of 0.8% of worker brood was infested per colony. Such infestations occurred in half of the colonies. The mite haplotypes did not differ significantly in the rate at which they infested worker brood at the two levels (colony and brood, Figure 3; Table S3).

When comparing the percentage of colonies with infested drone brood and with infested worker brood, one (Japan) and two (Samui and Malay) haplotypes of *V. destructor* and *V. jacobsonii*, respectively, differed significantly (Figure 3; Table S3). In these haplotypes, a larger percentage of colonies had drone brood infestations compared to worker brood infestations. Drone and worker brood infestation rates within colonies differed significantly in all haplotypes except *V. destructor* Korea (Table S4). When significant differences in infestation rates occurred, drone brood were more infested than worker brood (Figure 3a,b).

**Figure 4:**
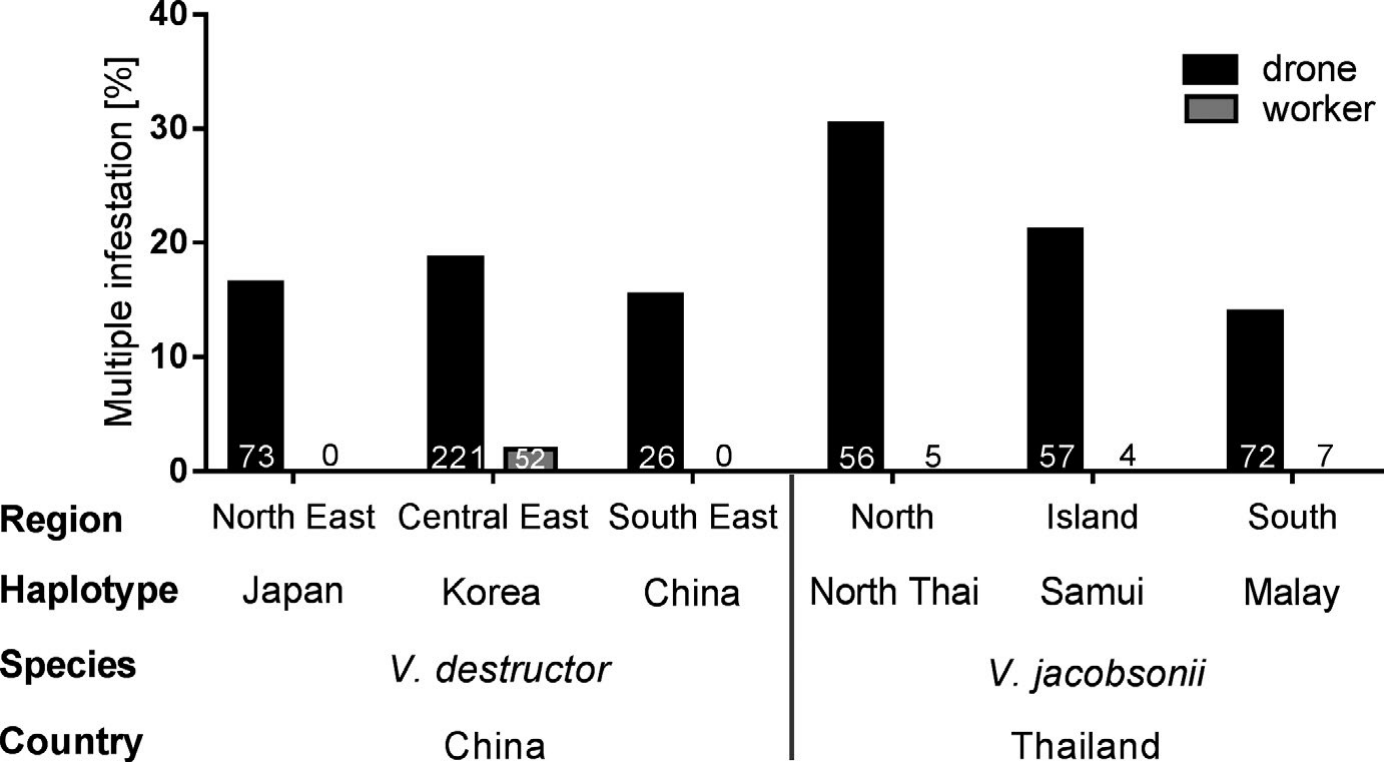
Percentage of *Apis cerana* drone and worker single cells infested by multiple foundresses of *Varroa destructor* and *Varroa jacobsonii* foundresses in China and Thailand, respectively. The percentages were calculated over all colonies within a population. There were no significant differences in the percentage of multiply infested cells between haplotypes (Table S3)

The percentage of drone cells infested with more than one foundress (i.e., with two to three) ranged between 11.5 for *V. destructor* China and 30.9 for *V. jacobsonii* North Thai haplotypes (Figure 4). Despite variations in the percentage of drone cells infested by multiple mites, no significant differences between haplotypes were detected (Table S3). A single worker brood cell was infested by more than one *V. destructor* foundress of the Korea haplotype in Central Eastern China (Figure 4).

### 3.2 Reproductive output

#### 3.2.1 Fertility

**Figure 5:**
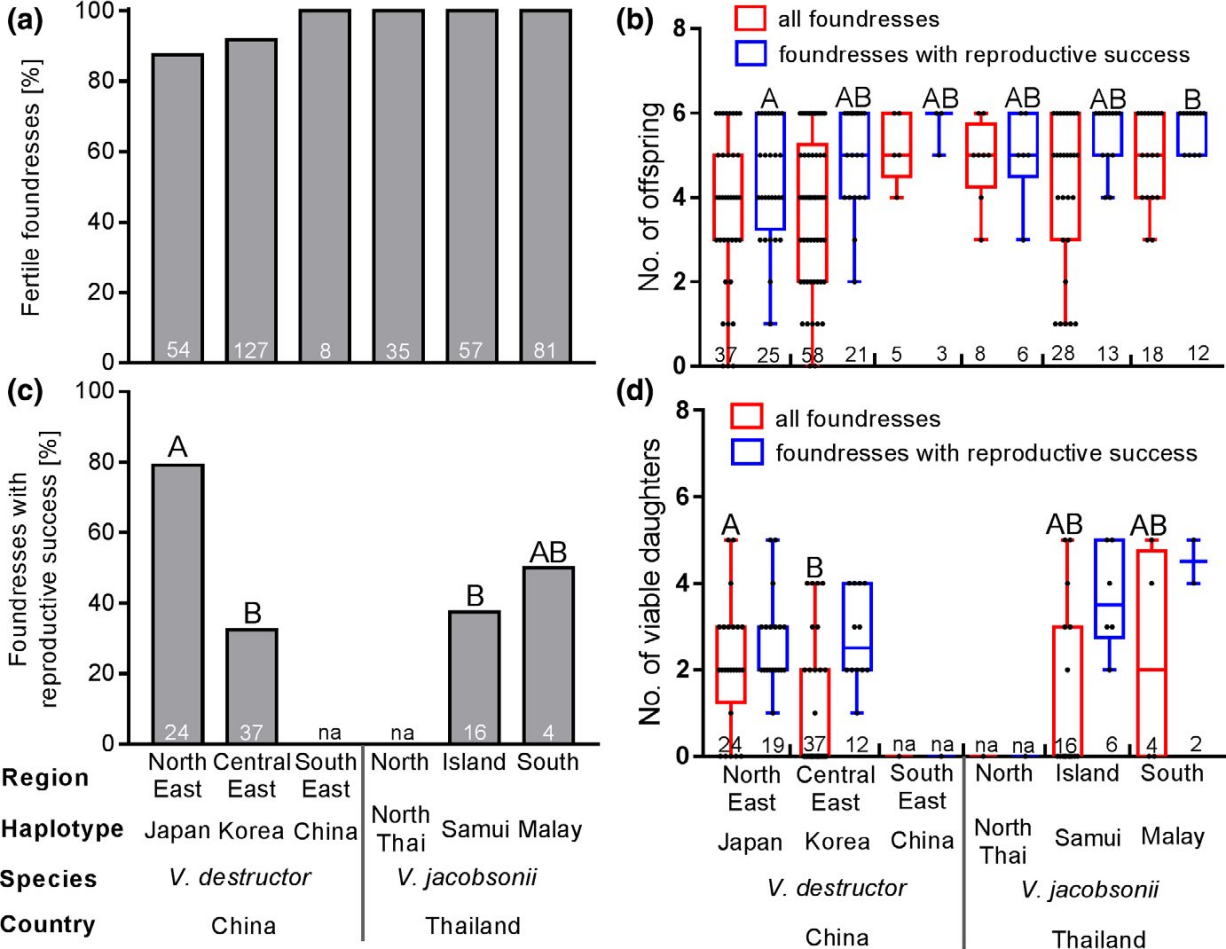
Reproduction of *Varroa destructor* and *Varroa jacobsonii* mites on drone brood of *Apis cerana*: (a) percentage fertile foundresses (i.e., with at least one offspring); (b) per capita fecundity (black dots = data points, median = horizontal line, box = 25/75% quartiles, whiskers = min–max range); (c) percentage foundresses with reproductive success (i.e., with at least one mated daughter); and (d) per capita number of viable daughters. The sample size is indicated within the bars. while “na” indicates groups with insufficient sample size. Different capital letters indicate significant differences obtained between haplotypes across species with the Fisher exact or Pearson chi‐square tests (percent fertile foundresses and foundresses with reproductive success) and with the Mann–Whitney *U* test (per capita fecundity and number of viable daughters), respectively, after FDR correction. FDR, false discovery rate

Most of the *V. destructor* (87.8%–100%) and *V. jacobsonii* (98.4%–100%) mites infesting drone brood at or beyond the prepupal stage were fertile. Although the overall comparison showed significant differences in fertility between haplotypes, none of the pairwise comparisons yielded significant differences after correcting for multiple comparisons (Figure 5a; Table S5).

#### 3.2.2 Fecundity

The average per capita fecundity varied between zero and six offspring when all foundresses (including sterile individuals) were considered and between one and six when only reproductively successful foundresses were considered. In both cases, there were significant differences among haplotypes. However, when all foundresses were considered, none of the pairwise comparisons yielded significant differences after correcting for multiple comparisons. When reproductively successful foundresses were considered, only those belonging to *V. destructor* Japan showed a significantly lower fecundity than *V. jacobsonii* Malay (Figure 5b; Table S5).

#### 3.2.3 Reproductive success

**Figure 6:**
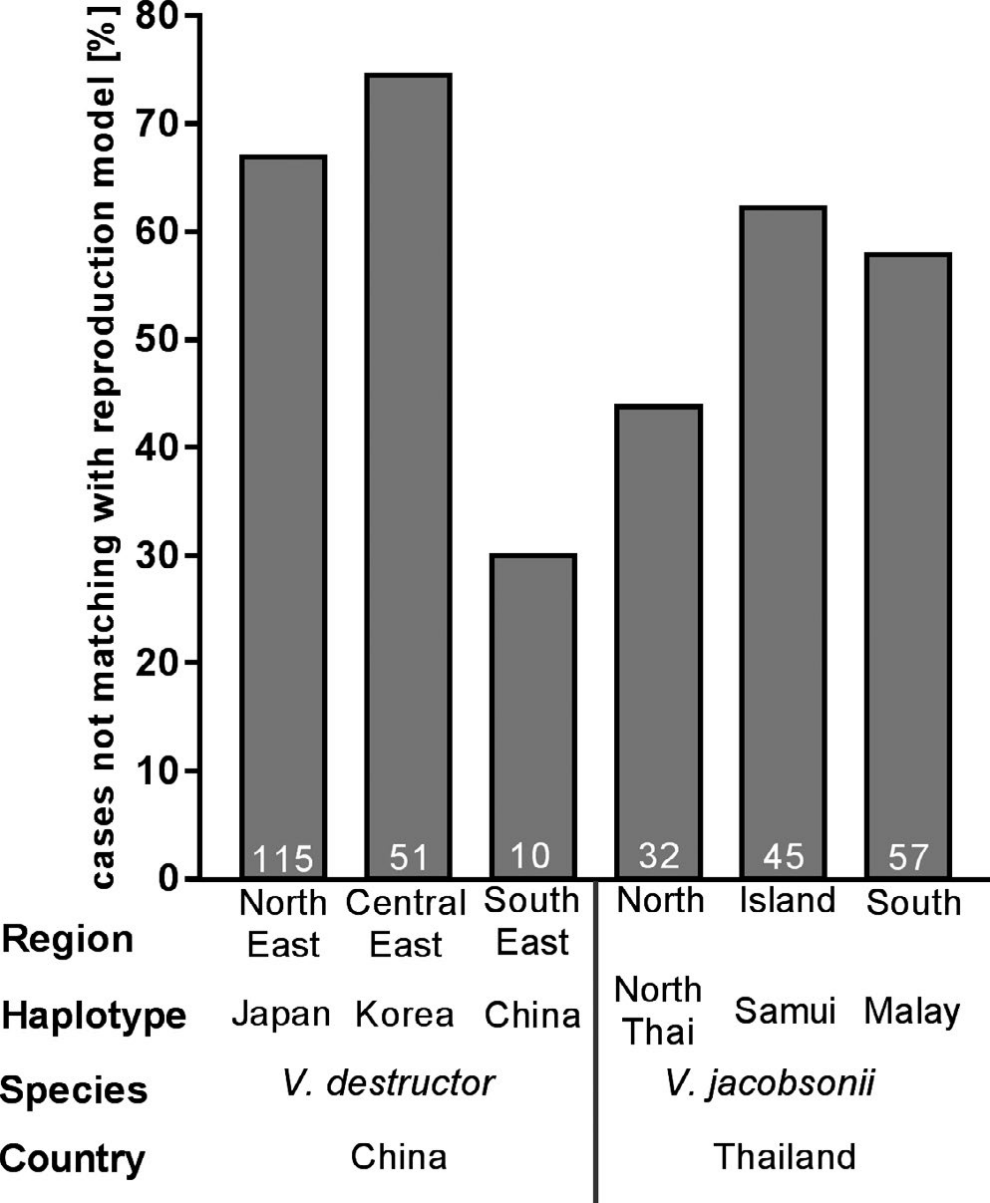
Percentage of cases showing divergence from the reproductive model for foundresses of each haplotype infesting drone brood of *Apis cerana*. The sample sizes are indicated within the bars. Asterisks indicate significant differences among all haplotypes using the Pearson chi‐square tests with FDR correction. *p < .05, **p < .01. FDR, false discovery rate

The percentage of foundresses with reproductive patterns not matching the model of reproduction (Table S2) varied from 30% to over 70% between haplotypes, but none of the pairwise differences between haplotypes were significant (Figure 6; Table S6). Deviations from the model originated in the frequent absence of sons (in 30.7% of the cells containing drone brood at and beyond the purple‐eyed stage, when male mites should be mature, Table S2) and from the occasional absence of mature daughters or of offspring of both sexes. As a result, only half of the foundresses on pre‐emergence adult hosts showed reproductive success. Two pairs of haplotypes differed significantly in the percentage of foundress reaching reproductive success: *V. destructor* foundresses of the Japan haplotype more often produced viable daughters than *V. destructor* Korea and *V. jacobsonii* of the Samui haplotype (Figure 5c; Table S5). Since many foundresses failed at reproducing successfully, a large difference in the number of viable daughters produced was observed when considering all foundresses or only foundresses that achieved reproductive success (Figure 5d). Only when considering all foundresses was a significant difference among haplotypes detected: *V. destructor* Korea produced significantly fewer viable daughters (mean ± *SD*, 0.9 ± 1.4) than the Japan haplotype (2.1 ± 1.5) (Table S5). Despite most differences between haplotypes being nonsignificant, the difference in average number of viable daughters between haplotypes could reach 1.2 individuals when considering all foundresses. This value reached 1.8 individuals when considering only foundresses with reproductive success.

Neither the correlation between fecundity and reproductive success (linear regression, R^2^ = 0.41, *F* = 1.39, *p* = .36) nor the correlation between the number of viable daughters produced by all foundresses and drone brood infestation rates were significant (linear regression, *R* ^2^ = 0.50, *F* = 1.99, *p* = .29).

#### 3.2.4 Reproduction in worker brood

Every developmental stage was represented in infested host worker brood, including those on which reproduction could be expected based on reproduction of the invasive lineage of *V. destructor* in *A. mellifera* (Dietemann et al., 2013). However, a single *V. jacobsonii* mite out of the 68 found in *A. cerana* worker brood had reproduced. This mite originated from the south of Thailand and infested a pre‐emergence worker. It had produced an egg and a deutonymph, but no sexually mature offspring.

## 4 DISCUSSION

Different *A. cerana* adult and brood infestation rates were found between the *Varroa* spp. haplotypes. However, differences in reproductive output were less frequent. Infestation of worker and drone brood of *A. cerana* brood types occurred, but, successful reproduction was restricted to mite foundresses infesting drone brood. Initiation of mite reproduction on worker brood was observed once, but did not lead to successful reproduction in the form of a mated daughter.

### 4.1 Features of coevolved *Varroa* spp.–*Apis* spp. systems

#### 4.1.1 Infestation rates

In China, noninfested *A. cerana* colonies were detected during the drone season, showing that these mites are not ubiquitous. The percentage of *A. cerana* colonies infested by *V*. *destructor* (40%–50%) in China appeared comparable to those reported by Zhou et al. (2015; up to 70%). This value is intermediate between that reported for *V*. *jacobsonii* (70%–100%, this study) and for *V*. *underwoodi* (0%–55%, 17% on average, Wang et al., 2019; measured simultaneously to the data presented in this study). Similar to the trend in percentage of infested colonies, *V. jacobsonii* showed significantly higher brood infestation rates than *V. destructor*. The range of drone brood infestation rates in this study (0.6%–8%) was in the lower range of those reported for *A. cerana* in the literature, reaching 80% in *V. jacobsonii* of Java for example (Koeniger et al., 1983; Rath, 1999), but higher than the range reported for *V. underwoodi*, which reached a maximum of 1% (Wang et al., 2019). Overall, these results suggest that a superior ability to spread within or among colonies is not linked to the virulence and invasiveness potential of a haplotype after host shift and indicate different local co‐evolutionary scenarios between hosts and parasites (Thompson, 2005).

Infestation rates could vary due to different mechanisms of tolerance or resistance to *Varroa* spp. of the host subspecies or populations. However, these differences in infestation parameters could also be affected by climate and season (García‐Fernández et al., 1995; Leza et al., 2016; Medina‐Flores et al., 2014; Ritter & de Jong, 1984; Tewarson et al., 1992). Unlike tropical Thailand, the sampled temperate areas of China have winters, which might result in shorter drone production periods and lower infestation levels. However, no data are yet available to confirm this hypothesis and comparisons within *V. destructor* contradict such trends, since the haplotype inhabiting the northernmost location with the harshest winters was significantly more prevalent at the brood level (Figure 3b). There might also be an influence of beekeeping management. While Chinese beekeepers occasionally remove sealed drone brood (Boes, 2010; Zeng et al., 2000), this is not occurring in Thailand, where *A. cerana* colonies are mostly kept in traditional hollow log hives with least management practices (Chantawannakul, 2018). This could partly explain the lower infestation rates measured in *V. destructor* compared to *V. jacobsonii*. Natural infestation rates in China should thus be confirmed by screening wild *A. cerana* populations or managed apiaries not subjected to drone brood removal. Since population size of the mites increases over the season (Tewarson et al., 1992), differences in sampling time can also bias the measures. Few studies measured infestation rates over time and most measures thus represent a snapshot (Tewarson et al., 1992). Repeated measures of both brood and adult worker infestation rates over time in various regions are therefore required to better understand the population dynamics of *Varroa* spp. in its original host.

When drone brood was present, adult infestations were only detected in a third of the colonies at rates below 1% in our study, indicating that most mites were in the reproductive stage in brood cells. No significant differences in infestations were found between the populations of the two species investigated, but this could be due to low sample size. Estimates of total colony infestation rates in various populations are also required to obtain a comprehensive picture of parasitic loads in the original host. However, there are only data available for one population (Rath & Drescher, 1990).

#### 4.1.2 Mite reproduction

Because the reproduction of *Varroa* spp. mites occurs in the actively heated honey bee brood (Tan et al., 2012), differences in individual reproductive output are less likely affected by external factor and thus more directly reflect the outcome of co‐evolutionary processes than infestation rates. The rare occurrence of significant differences in reproductive parameters of the various haplotypes (Figures 3 and 5; Table S5) suggests host (e.g., pupal development time) and/or parasite constraints (e.g., oogenesis cannot occur faster) to differentiation of reproductive output between haplotypes. Alternatively, sample sizes may have been too small to detect such differences. Because of this lack of difference, we can safely exclude that the ~10% of the *V. destructor* mites from the invasive Korea lineage or from the local Vietnam lineage in the northern Thai population (Dietemann et al., 2019) biased the comparison between haplotypes.

The fecundity of *V. destructor* and *V. jacobsonii* foundresses was poorly correlated to the number of viable daughters produced across haplotypes. In comparison with the number of offspring produced (i.e., fecundity), per capita number of viable daughters for all foundresses was low. The mechanisms underlying the low reproductive success despite high investment in oogenesis in *V. destructor*, *V. jacobsonii* (50%, this study) and *V. underwoodi*, (74%; Wang et al., 2019) as well as the differences or similarities in reproductive success between haplotypes (e.g., *V. underwoodi* produced a number of viable daughters similar to *V. destructor*; Wang et al., 2019) remain unknown to date (Rueppell et al., 2011).

Despite the general lack of significant differences in reproductive parameters between haplotypes, *V. jacobsonii* Malay produced on average up to 1.8 more viable daughters per capita than *V. destructor* Japan and Korea. Such ratio can have a large impact on population growth and colony infestation loads (five‐ and ten‐fold difference in population size after two and three generations, respectively). However, there was no significant correlation between the number of viable daughters and drone brood infestation rates of *V*. *destructor* and *V. jacobsonii*. This could be due to postemergence mechanisms affecting mite survival (e.g., grooming of the hosts; Fries et al., 1996; Peng et al., 1987) or to biased evaluation of infestation rates (see above). The factors underlying the relationship between these parameters should be identified for a better understanding of *Varroa* spp. population dynamics in the original host *A*. *cerana*.

### 4.2 Comparing coevolved and noncoevolved systems

Comparing infestation rates and reproductive output observed here with *V. destructor* infesting *A. mellifera* is challenging due to the paucity of data and to methodological differences. Moreover, infestation rates and reproductive output can vary over time (Anderson & Sukarsih, 1996; Garrido et al., 2003; de Mattos et al., 2016; Mondragón et al., 2005; Spivak & Reuter, 2001) and often only snapshots are reported during the drone season. Nevertheless, some patterns emerge (Table S7). The similarities in mite reproduction between *A. cerana* and *A. mellifera* suggest yet undescribed common resistance mechanisms (this study; Locke, 2016; Wang et al., 2019; Z Lin, S Wang, P Neumann, G Chen, P Page, L Li, F Hu, H Zheng & V Dietemann, unpublished data). Indeed, the number of viable daughters produced (0.5–2.5) appears to be in the same range for both hosts species (Table S7). Alternatively, but not mutually exclusive, physiological constraints of the mites may underlie these similarities. The most obvious difference between coevolved and noncoevolved taxa is the long known absence of reproduction in worker brood of *A. cerana* (Table S7). Although this trait may represent the central resistance mechanism of *A. cerana*, several populations of *A. mellifera* nevertheless survive *V. destructor* infestations despite such reproduction (Locke, 2016; Oddie et al., 2018; Table S7), indicating different evolutionary pathways to resistance in the two host species.

Even though high infestation rates of drone brood have been reported in *A. mellifera* (up to 51% in untreated colonies; Martin, 1995) and those of *A. cerana* being generally low, the highest values have actually been measured in *A. cerana* (Table S7). Together with generally lower fecundity and percentage of fertile mites infesting *A. mellifera* compared to *A. cerana* drones (Table S7), this suggests low selective pressure on the invasive lineage of *V. destructor* for maximizing reproduction on *A. mellifera* drones, possibly due their ability to reproduce on the more readily available worker brood in this host.

The frequency of infestations by several foundresses in *A. cerana* brood (ranging from 12% to 31% of the infested individuals) is high and appears to exceed those reported for *V. destructor* infesting *A. mellifera* (de Guzman et al., 2008; de Mattos et al., 2016; Spivak & Reuter, 2001; Strauss et al., 2016). These rates are likely determined by the ratio between parasite population size and amount of brood available. Given that mostly seasonal and less common drone brood is infested in *A. cerana*, a higher probability for multiple infestations can be expected. In brood cells hosting more than one foundress, male and female offspring of two or more mother mites can mate, thereby allowing for recombination (Beaurepaire et al., 2017) and contributing to the high genetic diversity of these populations (Dietemann et al., 2019; Z Lin, S Wang, P Neumann, G Chen, P Page, L Li, F Hu, H Zheng & V Dietemann, unpublished data). Multiple infestations can lead to hybridization between sympatric mite taxa, which are identified with increasing frequency and can affect the population structure and virulence of this parasite (e.g., Dietemann et al., 2019; Techer et al., 2020; Z Lin, S Wang, P Neumann, G Chen, P Page, L Li, F Hu, H Zheng & V Dietemann, unpublished data; H Zheng, S Wang, Y Wu, S Zou, V Dietemann, P Neumann, Y Chen, H Li‐Byarlay, C Pirk, J Evans, F Hu & Y Feng, unpublished data).

### 4.3 Maladaptations and traits putatively promoting host shifts

Even though the maximum *V. destructor* and *V. jacobsonii* infestation rate of worker brood in a host colony was low (0.8%), such infestations occurred in half of the colonies, indicating a widespread phenomenon. Interestingly, this was not the case for *V. underwoodi*, which rarely infested worker brood (one observation in 168 colonies, Wang et al., 2019). Infestations of *A. cerana* worker brood by *Varroa* spp. (range: 0%–13% of individuals infested; this study; Anderson & Sukarsih, 1996; Koeniger et al., 1981; Koeniger et al., 1983; Rath, 1999; Tewarson et al., 1992) could result from dysfunctions of host recognition and be maladaptive. Alternatively, infestations of worker brood may be adaptive. Because mites in cells are protected from grooming by adult hosts (Fries et al., 1996; Peng et al., 1987) and might benefit from feeding on larvae rather than on adults, their survival chances between the reproduction periods could increase (Boot et al., 1997). However, given that infestations of worker brood are adaptive, it should occur more frequently. In any case, the rare occurrence of native mites initiating reproduction in worker brood (this study; Boot et al., 1999; de Jong, 1988) is well in line with efficient resistance mechanisms in *A. cerana* (e.g., Harbo & Harris, 2005; Lin et al., 2016; Page et al., 2016). We observed the rare occurrence of offspring in a worker brood cell of *V. jacobsonii* in Southern Thailand. Despite parasitizing a pre‐emergence adult, the mite foundress had only produced sexually immature offspring and would not have been successful in producing a viable daughter. If a putative dysfunction of host type recognition in such foundresses also prevented them from distinguishing species‐specific brood cues, drifting between colonies of sympatric honey bee species (Dietemann et al., 2019; Z Lin, S Wang, P Neumann, G Chen, P Page, L Li, F Hu, H Zheng & V Dietemann, unpublished data) could give such atypical mites opportunities to successfully use a new host for reproduction. Interestingly and in support of this hypothesis, worker and drone brood infestation rates differed in all haplotypes except for the haplotype (Korea) from which the invasive lineage of *V. destructor* derived. Whether this trait predisposed this haplotype to shift host should be determined by identifying the mechanisms underlying host type choices, and whether mites able to infest and reproduce on atypical intraspecific hosts (workers and queens) are likely to reproduce on an alternative host species could be investigated with experimental infestations.

## 5 CONCLUSION

By screening the occurrence and reproductive status of mites in several populations of *A. cerana*, our study provides novel data on infestation rates of *A*. *cerana* brood and adults, as well as a quantification of the reproductive capacities of *V. destructor* and *V. jacobsonii* in their original host. A better understanding of the respective roles of parasite, host (Rueppell et al., 2010), and environmental factors in determining infestation rates, reproductive potential, and proximal mechanisms leading to atypical reproductive events would allow for a more accurate assessment of the risk posed by host shifts of *Varroa* spp. for managed and wild populations of *A. mellifera* and *A. cerana*. In addition to improving our understanding of host–parasite relationships, such knowledge would benefit programs aimed at selecting *A. mellifera* lineages resistant to *V. destructor* (e.g., Büchler et al., 2010; Guichard et al., 2020; Rinderer et al., 2010) by, for example, providing threshold values for infestations and reproduction levels permitting a host–parasite equilibrium.

## CONFLICT OF INTEREST

None declared.

## AUTHOR CONTRIBUTION

**Shuai Wang:** Data curation (equal); Investigation (lead); Methodology (equal); Visualization (equal); Writing‐original draft (equal); Writing‐review & editing (equal). **Zheguang Lin:** Data curation (supporting); Methodology (supporting). **Gongwen Chen:** Data curation (supporting); Methodology (supporting). **Paul Page:** Funding acquisition (supporting); Data curation (equal); Investigation (equal); Methodology (supporting); Project administration (supporting). **Fu‐Liang Hu:** Conceptualization (equal); Funding acquisition (equal); Project administration (equal); Supervision (equal). **Qingsheng Niu:** Resources (equal). **Xiaoling Su:** Resources (equal). **Panuwan Chantawannakul:** Project administration (equal); Supervision (equal); Conceptualization (equal); Writing‐reveiw &editing (equal). **Peter Neumann:** Conceptualization (equal); Funding acquisition (equal); Writing‐original draft (supporting); Writing‐review & editing (equal). **Huoqing Zheng:** Conceptualization (equal); Funding acquisition (equal); Resources (equal); Supervision (equal); Writing‐original draft (supporting); Writing‐review & editing (supporting). **Vincent Dietemann:** Conceptualization (equal); Funding acquisition (equal); Methodology (equal); Supervision (equal); Writing‐original draft (equal); Writing‐review & editing (equal).

## Supporting information

Table S1–S7

## Data Availability

The sampling locations, the number of colonies and cells that were sampled for the mites’ parasitic screening and the statistical analysis of infestation and reproduction parameters are available in the Supporting Information.
